# Are Individual Learning Experiences More Important Than Heritable Tendencies? A Pilot Twin Study on Placebo Analgesia

**DOI:** 10.3389/fpsyt.2019.00679

**Published:** 2019-09-18

**Authors:** Katja Weimer, Elisabeth Hahn, Nils Mönnikes, Ann-Kathrin Herr, Andreas Stengel, Paul Enck

**Affiliations:** ^1^Department of Psychosomatic Medicine and Psychotherapy, Ulm University Medical Center, Ulm, Germany; ^2^Department of Psychosomatic Medicine and Psychotherapy, Medical University Hospital Tübingen, Tübingen, Germany; ^3^Department of Psychology, Saarland University, Saarbruecken, Germany; ^4^Charité Center for Internal Medicine and Dermatology, Department for Psychosomatic Medicine, Charité-Universitätsmedizin Berlin, Corporate Member of Freie Universität Berlin, Humboldt-Universität zu Berlin, and Berlin Institute of Health, Berlin, Germany

**Keywords:** conditioning, expectation, heritability, learning, placebo analgesia, placebo effect, twins

## Abstract

**Objective:** Predicting who will be a placebo responder is a prerequisite to maximize placebo effects in pain treatment and to minimize them in clinical trials. First evidence exists that genetics could affect placebo effects. However, a classical twin study to estimate the relative contribution of genetic influences compared to common and individual environmental influences in explaining interindividual differences in placebo responsiveness has yet not been performed.

**Methods:** In a first explorative twin study, 25 monozygotic (MZ) and 14 dizygotic (DZ) healthy twin pairs (27.5 ± 7.7 years; 73% female) were conditioned to the efficacy of a placebo analgesic ointment with an established heat pain paradigm on their non-dominant arm. Placebo analgesia was then tested on their dominant arm. Furthermore, warmth detection thresholds (WDTs) and heat pain thresholds (HPTs) were assessed, and participants filled in questionnaires for the assessment of psychological traits such as depression, anxiety, optimism, pain catastrophizing, and sensitivity to reward and punishment. Their expectations were determined with a visual analog scale.

**Results:** There was a small but significant placebo analgesic effect in both MZ and DZ twins. Estimates of heritability were moderate for WDT only but negligible for HPT, the conditioning response, and placebo analgesia. Common environment did not explain any variance, and the individual environment explained the largest parts. Therefore, the placebo analgesia response can be seen as influenced by individual learning experiences during the conditioning procedure, whereas other variables assessed were not associated.

**Conclusions:** Compared to the individual learning experience, genetic influences seem to play a minor role in explaining variation in placebo analgesia in this experimental paradigm. However, our results are restricted to placebo effects through conditioning on pain in healthy volunteers and should be replicated in larger samples and in patients. Furthermore, potential gene–environment interactions should be further investigated.

## Introduction

Placebo effects are part of every medical intervention and should be used to maximize treatment effects in daily routine, but need to be minimized in randomized controlled clinical trials (RCTs) to estimate the “pure” drug effect (1, 2) that should exceed the placebo effect. Among the most challenging questions is the prediction of who will be a placebo responder or non-responder (3, 4). Placebo effects and responses are influenced by situational factors (5, 6), interact with personal factors (3) and prior experiences (7, 8), and are affected by the environment through explicit social (observational) learning of interventional effects (9, 10) and by an implicit social learning phenomenon called “placebo by proxy” (11, 12). Neither of these approaches has been able to allow the precise identification of placebo responders (1, 2).

Besides environmental or situational factors, studies following a molecular genetic approach have provided first evidence that genetic effects could influence placebo effects (13, 14). However, only a few studies investigated the association of genetic polymorphisms and placebo analgesia in healthy participants. Pecina and colleagues report that AA homozygotes compared to G carriers of the Mu-opioid receptor polymorphism (OPRM1 A118G) (15), as well as Pro/Pro homozygotes compared to Thr carriers of the fatty acid amide hydrolase (FAAH Pro129Thr) (16), showed higher placebo effects through verbal suggestions on pain induced through hypertonic saline. When placebo effects were induced through conditioning on thermal pain, Yu and colleagues found an association between Met allele carriers of the catechol-O-methyltransferase polymorphism (COMT Val158Met) and placebo analgesia (17). The latter is related to general dopamine release and has also been linked to placebo effects in irritable bowel syndrome (18) and major depression (19). Because of effects on different symptoms and in patients as well as in healthy participants, it seems to be an unspecific effect on placebo effects in response to the anticipation of rewarding situations. Subsequent studies aimed to replicate these findings with larger samples but did not find an association of the COMT genotype with placebo analgesia by verbal suggestion on thermal pain (20). Further studies combining polymorphisms of the before-mentioned genes show more promising but still inconclusive results. Aslaksen and colleagues found a significant placebo analgesic effect through verbal suggestion on thermal pain only in carriers of OPRM1 AA combined with COMT Met/Met and Val/Met alleles (21), whereas Colloca and colleagues found significant placebo analgesia in carriers of other combinations, namely, the combination of OPRM1 AA with FAAH Pro/Pro and the combination of COMT Met/Met with FAAH Pro/Pro, but not for OPRM1 AA with COMT Met/Met (Colloca et al., 2019). Furthermore, they found placebo effects in COMT Met/Val carriers independent of other combinations, and an interaction with the type of placebo induction through verbal suggestion or learning (22). Overall, results seem to be partly inconclusive, but influencing factors such as the type of pain stimuli and placebo procedure have only seldom been considered.

However, the so-far identified candidate genes and polymorphisms show rather small effects and neither allow reliably predicting placebo responders across clinical conditions and experimental paradigms nor can distinguish between genetic and environmental contributions to the placebo effect (23, 24). Here, quantitative behavioral genetic methods such as the classical twin design (CTD) are traditionally used to disentangle and estimate the relative contribution of genetic and environmental influences in explaining interindividual differences in human behavior. By comparing the observable similarities of monozygotic (MZ) and dizygotic (DZ) twins—who share 100% (MZ) and respectably 50% (DZ) of their segregating genes—the relative importance of genetic influences can be inferred in the sense that they are assumed to be important when MZs are twice as similar as DZs. The centerpiece of the CTD represents the heritability estimate (H), which describes the proportion of the total variance explained by the genetic variance. The remaining part of the variation can then be attributed to environmental influences from different kinds of sources (e.g., family, individual experiences, situational conditions) typically subdivided into common (leading to similarity between family members) and individual (leading to differences between family members) environmental influences. Although twin studies have been conducted successfully for more than 50 years, they are lacking so far in placebo research to assess the variance that could be explained by genetic, common, and individual environmental components (25–27).

Only few studies in healthy twins have investigated pain sensitivity and analgesic drug responses. Nielsen et al. (28) investigated pain sensitivity and found less evidence for both genetic and common environmental factors in an experimental study with 53 MZ and 39 DZ twin pairs: Genetic factors could only explain 7% and 3% of the variance in cold pressor and heat pain, respectively, and environmental factors explained only 5% and 8% of variance, respectively. In contrast, Angst et al. (29) employed 81 MZ and 31 DZ healthy twin pairs in an experimental study and found a significant heritability for cold pressor pain tolerance (explaining 49%) and a significant interaction of genetic and environmental effects for heat and cold pressor pain thresholds (explaining 24% and 32%, respectively). After infusion of alfentanil, a µ-opioid agonist, they found significant heritability for the analgesic effect in cold pressor pain thresholds (60%) and a familial effect on cold pressor pain tolerance (30%). Unfortunately, the results of the placebo arm were not reported.

Placebo analgesia, i.e., the pain reduction after the application of an inert treatment, is the best investigated paradigm to study the mechanisms underlying the placebo effect (conditioning, expectation, social learning). This has been tested with different pain stimuli (e.g., heat pain) and in healthy volunteers as well as in pain patients. An established heat pain paradigm was employed to induce a conditioned placebo analgesic effect (7, 30–32). The classical twin study design is an established methodology to differentiate between genetic and environmental factors (25, 27).

Our study combines these two approaches—conventional placebo analgesia stimulation with a heat pain paradigm and a classical twin study design—to explore the relative influence of genes and the environment on the placebo response in experimental pain in healthy twins for the first time. Based on the mixed results reported by previous studies using different experimental designs, we would like to reexamine the question whether differences in placebo effects actually show a heritable component—as should be expected based on the first law of behavior genetics postulating that everything is heritable (33)—in contrast to an equally conceivable assumption of primary environmental learning influences as a source of individual differences in placebo responses given the strong learning component of analgesia responses. Furthermore, our results aim to stimulate further studies with twins to address open questions in the field of heritability and genetic influences on placebo effects.

## Methods

### Participants

A community sample of 40 MZ and DZ healthy twin pairs were recruited through the database of HealthTwiSt GmbH, Germany (34), and by email at the University of Tübingen, Germany. Inclusion criteria were: between 18 and 60 years old, raised together, fluent in German, and participation of both twins in the study. They were excluded when at least one twin had acute or chronic diseases of the skin, pain disorders, disorders of the cardiovascular system, psychiatric disorders, other acute or chronic conditions or medication intake that affects pain sensitivity or reaction times. They were asked to refrain from drinking alcohol or taking medication for at least 24 h before the experiment. Inclusion and exclusion criteria were checked through online questionnaires and by the investigator before the experiment. One twin pair was excluded due to technical problems during testing.

All participants were included after written informed consent only and received monetary rewards for their participation in this study. This study was approved by the Ethical Review Board of the University of Tübingen (project no. 814/2015BO1) and was conducted in accordance with the Declaration of Helsinki.

### Zygosity Assessment

Zygosity was assessed based on questions about previous genetic zygosity tests, intrapair resemblance, and confusion by strangers. This has been shown to reliably distinguish between MZs and DZs (27, 35, 36). Ten MZs and one DZ reported that genetic tests were performed. A zygosity score between 0 (high dissimilarity) and 20 (high resemblance) was calculated and compared to twins’ own knowledge or opinion about their zygosity. This score significantly distinguished between MZs and DZs (11.6 ± 1.7 vs. 3.4 ± 3.7, respectively, *t*(76) = 13.44, *p* < .001) and confirmed the twins’ own information.

### Study Design

**Figure 1:**
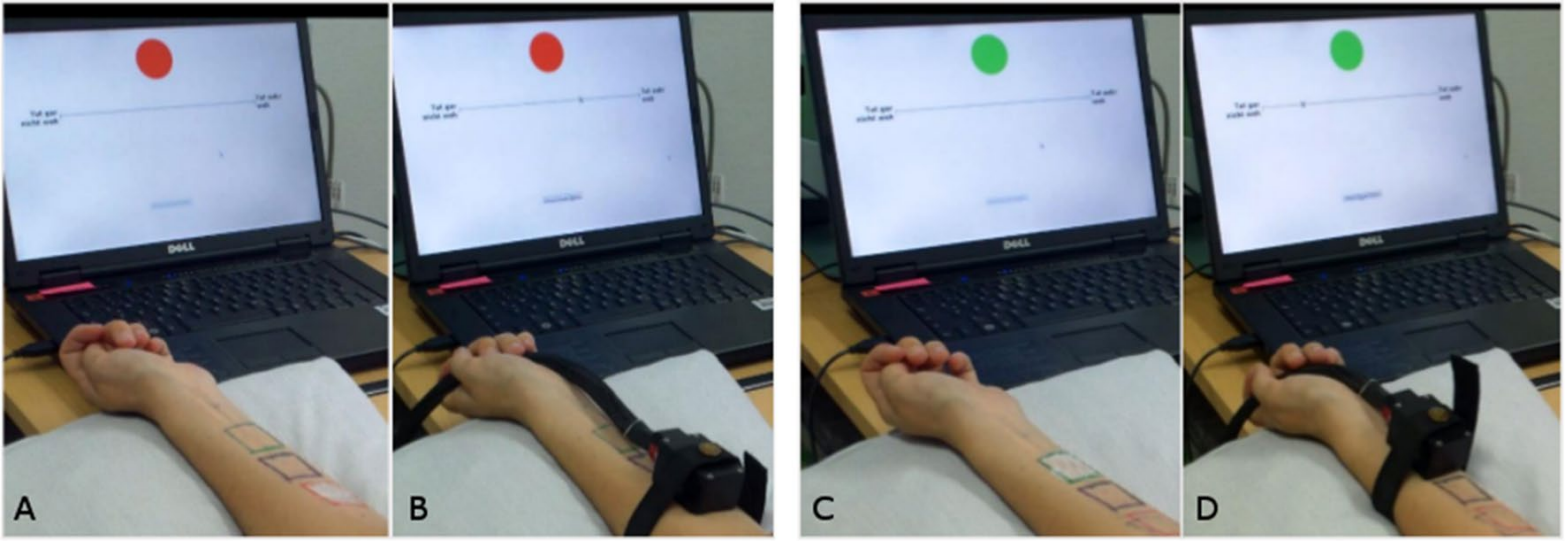
Experimental setup. **(A)** Ointment placed on the first area marked in red and computer screen with a visual analog scale (VAS). Participants were reminded that this is the control ointment. **(B)** After 5 min and removal of the ointment, the thermode is placed on the red field and heat pain stimuli started. **(C)** Ointment placed on the second area marked in green and computer screen with VAS. Participants were reminded that this is the effective analgesic ointment. **(D)** After 5 min and removal of the ointment, the thermode is placed on the green field and heat pain stimuli started.

All participants took part in the study on a single occasion between 11.00 a.m. and 6.30 p.m. They were informed about the study aims as being effects of genetics and implicit learning on pain sensitivity and perception. After written informed consent, inclusion and exclusion criteria were double-checked through a short anamnesis questionnaire by the experimenter. Experiments were performed on both volar forearms, beginning with the non-dominant arm (arm 1) followed by the dominant arm (arm 2) of the participant. This order was chosen so that participants could use a computer mouse and press buttons with their dominant hand as usual. On both arms, the warmth detection threshold (WDT), heat pain threshold (HPT), and testing of two ointments, a control and a placebo ointment, were performed. Therefore, three squares of 3 × 3 cm for the positioning of a thermode were painted on the forearm: a black one in the middle of the forearm, and a green and a red one above and below, respectively (**Figure 1**). Distal and proximal positions of the green and red squares were randomized between twin pairs but kept constant within one pair. Participants were conditioned for the effectiveness of an inert ointment application on arm 1, and placebo analgesia was tested on arm 2. Between tests on both arms, participants filled in questionnaires for around 30 min.

All heat stimuli were applied with a thermode (TSA-II, Medoc Ltd., Ramat Yishai, Israel), which can apply temperatures between 0°C and 50°C on a square of 3 × 3 cm. Baseline temperature was set to 32°C for all tests.

### Outcome Measures and Conditioning Procedure

For the assessment of thresholds, the thermode was placed on the middle, black square. The assessment of thresholds was performed according to the quantitative sensory testing (QST) protocol (37). The temperature of the thermode increased by 0.5°C/s until the participant pressed a mouse button when she or he felt an increase of the temperature for the first time. Then the temperature decreased to the baseline automatically with a return rate of 1°C/s. The mean of three assessed temperatures was calculated as WDT. For the assessment of HPT, the temperature of the thermode increased by 1°C/s until the participant pressed a mouse button when the stimulus was perceived as painful for the first time. The temperature decreased to the baseline with a return rate of 10°C/s. The mean of three assessed temperatures was calculated as HPT.

Participants were familiarized with the rating of heat stimuli on a visual analog scale ranging from 0 (not painful at all) to 10 (extremely painful) by presenting three heat stimuli equal to the HPT and 1°C above and below HPT, respectively. Afterwards, eight stimuli of 10 s (∼1.5 s ramp-up and ∼1.5 s ramp-down) ranging between −1°C and +2°C in pseudo-randomized order were applied and rated by the participants. Temperatures according to a rating of 2 and 5 on the VAS were calculated by means of linear regression analyses and were used as conditioning temperature (VAS2) and as test temperature (VAS5). For conditioning on arm 1, an inert ointment (Base Cream DAC, Bombastus-Werke AG, Freital, Germany) was applied to the red square for 5 min and removed, and then eight heat stimuli of 10 s (with ∼1.5 s ramp-up and ∼1.5 s ramp-down) according to VAS5 were applied to this square and rated by the participant. Afterwards, an inert application of a topical analgesic cream (EMLA cream, AstraZeneca GmbH, Wedel, Germany) was applied to the green square for 5 min and removed, and then eight heat stimuli of 10 s (with ∼1.5 s ramp-up and ∼1.5 s ramp-down) according to VAS2 were applied to this square and rated by the participant. Conditioning was supported by the information that the first ointment is inert and the second ointment is EMLA, a potent analgesic ointment. Furthermore, during application and ratings, a green or red circle, respectively, was shown on a monitor. Means of the eight ratings as well as the difference between these means were calculated and reported as conditioning response. Our application of EMLA was ineffective, as studies have shown that EMLA comes into effect after application on the skin after at least 30 to 60 min (38–41). Using EMLA had the advantage that deception of participants was reduced to a minimum, as they were told honestly that it is an effective analgesic ointment. Placebo testing was performed on arm 2 through application of the inert and the EMLA ointments in the same way as on arm 1, but with the difference that on both squares, eight heat stimuli according to VAS5 were applied. Information and colored circles were provided like in the conditioning procedure. Means of the eight ratings as well as the differences between these means were calculated and reported as placebo analgesia.

### Questionnaires

Studies have shown that placebo analgesia could be influenced by individual psychological characteristics (3, 42) such as optimism (43), the extent of depressive or anxious symptoms also in healthy individuals (44), pain catastrophizing, as well as expectations concerning the effectiveness of treatment (42). Furthermore, it has repeatedly been hypothesized that reward sensitivity could affect placebo analgesia (42, 45). To analyze such factors as predictors of placebo analgesia, the following questionnaires were assessed: scales for depression and anxiety of the Patient Health Questionnaire (PHQ) (46), Life Orientation Test—Revised version (LOT-R) (47), Pain Catastrophizing Scale (PCS) (48), and Sensitivity to Punishment and Sensitivity to Reward questionnaire (SPSR) (49).

Expectancy was assessed by the question, “How effectively do you think the treatment will reduce the heat pain?” and rated by participants on a VAS from 0 (no effect) to 10 (strong effect). In order that participants not become suspicious about the study design, expectancy was assessed during each application time but analyzed only for the relevant placebo testing (EMLA on arm 2).

### Statistical Analyses

Phenotypic statistical analyses were performed with IBM SPSS Statistics for Windows, Version 25.0 (IBM Corp., Armonk, NY). Significance level was set at *p* < .05 for all analyses.

Sample size was calculated for the correlation of the main outcome, placebo analgesia, between a twin and his or her co-twin, for which a sample size of n = 67 was sufficient (with r = .3, alpha = .05, power = .80), as calculated with G*Power Version 3.1.9.2 (50). Normal distribution of variables was assessed with Shapiro–Wilk and Kolmogorov–Smirnov tests and visual inspection of data with normal quantile–quantile plots. Differences between groups were analyzed with Student’s t-tests. Conditioning response as well as the placebo analgesic effect were tested with paired t-tests for the rating of the control ointment and the rating of the inert EMLA ointment.

In our sample, pain-related outcome variables, reported in **Table 1**, did not differ between female and male participants. Furthermore, handedness did not affect any of the pain-related outcomes reported in **Table 1** (72 participants were right- and 6 were left-handed). Twin data were arranged according to the order of birth, and outcome variables reported in **Table 1** did not differ, neither between firstborn and second-born twins nor between MZ and DZ twins.

**Table 1:** Pain-related outcome measures in MZ and DZ twin pairs (reported as mean ± standard deviation) and intraclass correlations (reported as ICC coefficients and 95% CI).

Parameter	Monozygotic twin pairs (n = 25)			Dizygotic twin pairs (n = 14)		
	Twin 1	Twin 2	ICC [95% CI]	Twin 1	Twin 2	ICC [95 % CI]
**Arm 1**						
Warmth detection threshold (°C)	33.8 ± 1.3	33.7 ± 0.8	.452** [.077 to .715]	34.3 ± 1.3	33.5 ± 0.6	.045 [−.480 to .546]
Heat pain threshold (°C)	43.5 ± 2.8	43.3 ± 2.4	−.063 [−.441 to .333]	43.4 ± 2.8	43.1 ± 2.7	.247 [−.306 to .675]
Rating control (VAS)	5.0 ± 1.3	4.7 ± 1.6	.000 [−.388 to .388]	4.9 ± 1.6	5.4 ± 1.1	−.371 [−.743 to .177]
Rating EMLA (VAS)	3.1 ± 1.1	2.6 ± 1.2	.171 [−.233 to .524]	2.9 ± 1.4	2.9 ± 1.7	.137 [−.405 to .608]
Conditioning response (ΔVAS)	−1.9 ± 1.1	−2.0 ± 1.2	−.366 [−.660 to .026]	−2.0 ± 2.2	−2.4 ± 1.5	−.175 [−.632 to .372]
**Arm 2**						
Warmth detection threshold (°C)	34.0 ± 1.0	33.7 ± 0.8	.458** [.085 to .719]	34.1 ± 1.0	33.6 ± 0.6	−.150 [−.607 to .394]
Heat pain threshold (°C)	43.1 ± 3.2	43.1 ± 2.5	.043 [−.351 to .424]	42.3 ± 2.6	41.9 ± 2.7	−.103 [−.586 to.434]
Rating control (VAS)	5.2 ± 1.6	4.6 ± 1.6	.028 [−.365 to .411]	5.1 ± 1.8	5.9 ± 1.2	−.217 [−.658 to .334]
Rating EMLA (VAS)	4.7 ± 1.6	4.2 ± 1.6	.169 [−.235 to .523]	4.4 ± 1.6	5.3 ± 1.6	−.423 [−.770 to .116]
Placebo analgesia (ΔVAS)	−0.5 ± 0.9	−0.4 ± 0.6	.125 [−.277 to .489]	−0.7 ± 1.2	−0.6 ± 0.9	−.489 [−.802 to .033]

**p < .01. MZ, monozygotic; DZ, dizygotic; ICC, intraclass correlation coefficient; VAS, rating on a visual analog scale from 0 to 10.

All behavioral genetic models were fitted using the OpenMx package (51). Prior to estimating genetic and environmental influences as well as correlations within twin pairs [assessed by intraclass correlations (ICCs)], all variables were residualized for age, age squared, sex, and interaction effects between age and sex by multiple regression procedures, as the perfect correlation for age and sex in twin pairs can inflate twin similarities (52).

Behavioral genetic research is based on the simple rationale that genetic influences are relevant for a specific trait when biological relatives are more alike than unrelated individuals. On the other side, family members sharing relevant environmental factors should be more alike than family members and unrelated individuals who do not share this environment. By comparing MZ and DZ twins, who share family environmental influences but differ in their genetic relatedness, these different sources of variation in a given trait, e.g., placebo response, can be distinguished and estimated. To estimate the relative contribution of genetic and environmental influences for individual differences in all relevant factors, we performed univariate genetic modeling decomposing the phenotypic variation into variation due to genetic influences (labeled as A for additive genetic variance) and environmental influences, which are subdivided into common environmental influences (labeled as C) and individual environmental influences (typically labeled as E including measurement error) (so-called ACE model). Based on MZ and DZ resemblances, different expectations about genetic and environmental influences can be formulated: If the within-MZ correlation is greater than the DZ correlation, genetic influences can be assumed. A high correlation within both MZs and DZs indicates common environmental influences (shared between family members), while low correlations within both MZs and DZs, as well as any differences between MZ twins growing up in one family, can be attributed to individual environmental effects and measurement error. Overall, it is important to note that genetic and common environmental influences increase intrapair twin similarity, whereas the individual environment decreases it.

A detailed description of the model fitting approach and estimation of heritability can be found elsewhere (53). Due to the limited sample size and hence power considerations, we focused on the results for the full model given that the exclusion of any genetic or environmental effect may result in biased estimates of the remaining factors in the model, even if the removed factor was not significant (54).

Assumptions of this model are that 1) theoretically, MZs share 100% of their segregating genes, while DZs share 50%; 2) both MZs and DZs raised together share 100% of their common environment; and 3) all other effects such as individual environmental influences, individual learning experiences, and measurement errors contribute to differences within twin pairs. Furthermore, the applied genetic model relies on a number of prerequisites (for details, see 55), such as that twins are generalizable to the rest of the population and that genetic and environmental influences are independent from one another.

Further predictors of placebo analgesia, such as the conditioning response (regarded as the individual learning experience), the co-twins’ placebo analgesia (regarded as an estimate of aggregated familial effects), pain sensitivity of test arm (HPT on arm 2), expectancy, and psychological variables, were analyzed with Pearson’s correlations, and *p* values are reported. Due to the exploratory nature of this study and as all predictors were reasonably chosen based on previous results, unadjusted *p* values are reported, but also, results when *p* values are adjusted for multiple testing according to Benjamini and Hochberg [false discovery rate (FDR)] (56). We planned to include significant predictors in a linear regression analysis to account for multiple predictors at the same time, but as the conditioning response was the only significant predictor, regression analysis was obsolete.

## Results

### Study Population and Outcome Measures

Of 39 twin pairs, 25 were MZ (19 female, 6 male) and 14 were DZ (7 female, 2 male, and 5 opposite sex). MZs were 28.3 ± 8.2 years old, and DZs were 25.9 ± 6.7 years old (*t*(37) = 0.93, *p* = .36).

Calculated test temperatures according to a VAS of 5 were 45.8 ± 2.1°C for MZ and 46.1 ± 2.3°C for DZ and did not differ between MZ and DZ (*t*(76) = −0.59, *p* = .56). Calculated test temperatures according to a VAS of 2 were 43.4 ± 2.3°C for MZ and 43.6 ± 2.5°C for DZ and did not differ between MZ and DZ (*t*(76) = −0.40, *p* = .69).

### Warmth and Pain Sensitivity

WDT significantly correlated within MZ twins on both arms, but not between DZ twins. There were nearly no correlations of HPT between MZ twins on both arms; however, there was a low correlation between DZs on arm 1, but no correlation on arm 2 (**Table 1**).

### Conditioning Response and Placebo Analgesia

Among all participants, there was a significant conditioning response, with a mean pain reduction on the VAS from 4.9 ± 1.4 to 2.9 ± 1.3 (*t*(77) = 12.38, *p* < .001, 20% of VAS) on arm 1, and a significant placebo analgesic effect, with a mean pain reduction from 5.1 ± 1.6 to 4.6 ± 1.6 (*t*(77) = 5.25, *p* < .001, 5% of VAS) on arm 2. Of all participants, 68% reported a pain reduction, whereas 32% reported no difference or an increase in pain on arm 2. Furthermore, both effects were significant within MZ (*t*(49) = 11.64, *p* < .001 and *t*(49) = 4.04, *p* < .001, respectively) and within DZ twins (*t*(27) = 6.31, *p* < .001 and *t*(27) = 3.39, *p* = .002, respectively) when analyzed separately (**Table 1**).

### Genetic, Common, and Individual Environmental Contributions to Pain-Related Outcomes and Placebo Analgesia

Twin resemblances (reported as ICCs) and their respective confidence intervals are shown in **Table 1**. Except for WDT, the pattern of ICCs between MZ and DZ twin pairs did not suggest genetic influences to be an important source of variation. In accordance, the results of behavioral genetic model fitting (shown in **Table 2**) showed that estimates of heritability were extremely low or negligible. For WDT, the performed ACE model included heritability estimates of 34% (arm 1) and respectively 38% (arm 2), with the remaining variance explained by individual environmental influences (66% arm 1 and 62% arm 2). For all other traits, individual environmental influences were the major source of variation explaining between 85% and 100% of the variation.

**Table 2:** Standardized estimates of heritability (h^2^), common (c^2^) and individual environmental (e^2^) effects on pain-related outcomes, conditioning response, and placebo analgesia.

Parameter	Heritability	Common environment	Individual environment
**Arm 1**			
Warmth detection threshold (°C)	.336 [.000–.627]	.000 [.000–.442]	.664* [.373–1.00]
Heat pain threshold (°C)	.000 [.000–.344]	.027 [.000–.332]	.973* [.668–1.00]
Rating Control (VAS)	.000 [.000–.295]	.000 [.000–.219]	1.00* [.705–1.00]
Rating EMLA (VAS)	.161 [.000–.533]	.000 [.000–.373]	.839* [.467–1.00]
Conditioning response (ΔVAS)	.000 [.000–.182]	.000 [.000–.126]	1.00* [.818–1.00]
**Arm 2**			
Warmth detection threshold (°C)	.383 [.000–.676]	.000 [.000–.394]	.617* [.324–1.00]
Heat pain threshold (°C)	.000 [.000–.000]	.000 [.000–.000]	1.00* [1.00–1.00]
Rating Control (VAS)	.004 [.000–.354]	.000 [.000–.000]	.996* [.646–1.00]
Rating EMLA (VAS)	.050 [.000–.410]	.000 [.000–.274]	.950* [.590–1.00]
Placebo analgesia (ΔVAS)	.000 [.000–.357]	.000 [.000–.194]	1.00* [.643–1.00]

*p < .05. VAS, rating on a visual analog scale from 0 to 10.

### Prediction of Placebo Analgesia

**Figure 2:**
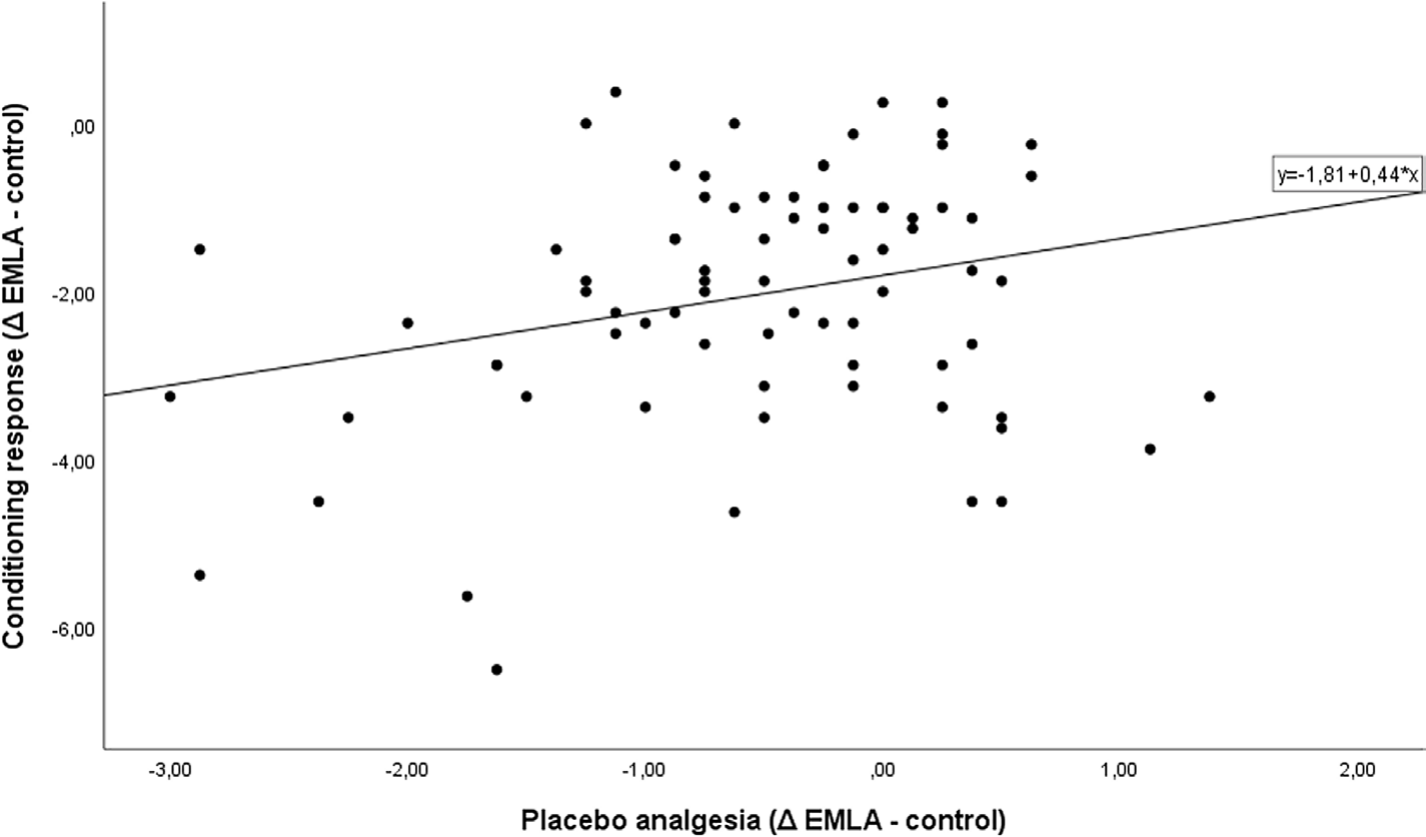
Correlation between conditioning response and placebo analgesia.

To further explore influences on the estimated high individual environmental effect on placebo analgesia, predictors were analyzed (**Table 3**). Placebo analgesia significantly correlated positively with the conditioning response only (*r* = .265, *p* = .019) (**Figure 2**) but not with any of the other predictors. The conditioning response itself was significantly associated with pain sensitivity (*r* = −.239, *p* = .035), the test temperature used (*r* = −.493, *p* < .001), pain catastrophizing (*r* = .229, *p* = .043), and expectancy (*r* = −.249, *p* = .028).

**Table 3:** Correlations between placebo analgesia, ratings of control and EMLA ointments, conditioning response, and predictors (reported as Pearson’s correlation coefficients r; n = 78).

Parameter	Placebo analgesia		Conditioning response	
	***r***	***p***	***r***	***p***
Conditioning response	.265	.019		
Rating control (VAS, arm 2)	−.244	.031	−.456	<.001
Rating EMLA (VAS, arm 2)	.300	.008	−.305	.007
Placebo analgesia co-twin	−.129	.260	−.039	.736
Pain sensitivity (HPT arm 2)	<.001	.997	−.239	.035
Test temperature (acc. VAS-5)	−.054	.640	−.493	<.001
Depression (PHQ)	−.114	.325	.136	.239
Anxiety (PHQ)	−.105	.364	−.013	.913
Optimism (LOT-R)	.089	.438	.125	.274
Pain catastrophizing (PCS)	−.061	.594	.229	.043
Sensitivity to punishment (SPSR)	−.060	.602	.056	.627
Sensitivity to reward (SPSR)	−.062	.591	.179	.117
Expectancy	−.034	.765	−.249	.028

HPT, heat pain threshold; PHQ, Patient Health Questionnaire; LOT, Life Orientation Test; PCS, Pain Catastrophizing Scale; SPSR, Sensitivity to Punishment and Sensitivity to Reward questionnaire.

Placebo analgesia also significantly correlated negatively with the rating of the control ointment and positively with the inert EMLA ointment, as placebo analgesia was calculated as the difference between them. The ratings of the ointments on the test arm (arm 2) were significantly negatively correlated with the conditioning response: the better the conditioning response (more negative), the higher the ratings on the test arm (**Table 3**).

When *p* values were adjusted for multiple testing, there was no significant correlation between placebo analgesia and the predictors, but the conditioning response was still significantly associated with the ratings of the control and EMLA ointments (*p* < .001 and *p* = .028, respectively) and with the test temperature used (*p* < .001).

## Discussion

To the best of our knowledge, this is the first study with MZ and DZ twins in placebo research and estimating the variances explained by heritability, common environmental, and individual learning components of placebo analgesia. For this purpose, we used an established conditioning paradigm with heat pain stimulation and inert ointment applications to induce placebo analgesia. Furthermore, we examined the role of these components (genetics, common and individual environment) in heat pain-related measures such as WDT, HPT, temperature ratings, and the conditioning response, and their association with placebo analgesia. We explored the effects of psychological traits on placebo analgesia. Finally, this pilot study shows open questions in the field of heritability and genetic influences, which should be further investigated.

WDT as well as HDT were assessed according to the quantitative sensory testing protocol and lie within the reported reference values as reported by Rolke and colleagues (37). With the conditioning paradigm used, participants reported a significant pain reduction of 5% on the VAS when the placebo ointment compared to the control ointment was applied (on arm 2), and 68% of participants reported reduction of pain. Reported placebo analgesia is highly variable between published studies; for example, Eippert et al. found a pain reduction of 23% (30), and Wager et al. detected 22% (31), whereas Wrobel et al. found placebo effects of around 4% in adults and 7% in children (at least according to the figure presented, as no data were mentioned) (32). The latter had the most similar study design to our study. Accordingly, we found comparable placebo analgesic effects. The placebo responder rate of 68% is comparable to the rate of 72% reported by Wager et al. (31). Differences in placebo analgesia could be due to differences in study designs, e.g., how many conditioning trials were performed, if conditioning and placebo testing were performed on the same day, and test temperatures.

Our study results show poor to fair (57) correlations within MZ twin pairs for WDT only, whereas correlations in HPT, ratings of ointments, conditioning responses and placebo analgesia were even lower and not significant in MZs as well as DZs. The pattern of low intrapair correlations in both MZ and DZ twins points to the fact that there is a low influence of heritability as well as common environmental components, which both are supposed to increase similarity between twins, and that the individual, nonshared environment may play a major role. The latter contributes to the dissimilarity of twins. Estimates of heritability (h^2^) and common (c^2^) and individual (e^2^) environmental effects confirm the pattern found: moderate heritability was found for WDT on both arms only, whereas heritability of ratings of heat pain stimuli after ointment application varied between ointments and arms but was very low. Regarding the conditioning response and our main outcome, placebo analgesia, individual environmental influences explained 100% of the variation. To further investigate individual factors influencing placebo analgesia, questionnaires assessing traits that were previously found to affect placebo or nocebo effects (3, 42) were collected. In this study, placebo analgesia is correlated with ratings of ointments (not surprisingly, as it is calculated from those) and with the conditioning procedure as the only significant predictor. The conditioning procedure in turn is correlated with HPT as a measure of pain sensitivity, test temperature, and pain catastrophizing.

Results of our study show that genetics may play a role in WDT, but the individual environment plays a more important role in placebo analgesia than genetics or the common environment of twins. The genetic influence in WDT could be explained by a stronger involvement of physiology than cognitive and emotional appraisal, as the early detection of warmth implies low danger for tissue damage. It is well known that the perception of clinical and experimental pain is not only determined by physiology—*via* neuronally mediated nociception—which may be under genetic control, but is also influenced by cognitive and affective appraisals. The latter are subjective evaluations of pain signaling, which are influenced by learning from previously experienced situations (58, 59). In contrast to WDT, appraisal and learning mechanisms become more important with stimuli above the pain threshold, as for the induction of placebo analgesia. Such individual learning experiences have already been shown to play an important role in placebo analgesia in other experimental studies (1, 7, 30, 32) as well as in clinical analgesic trials (60).

The individual learning experience as induced by conditioning was in turn affected by other factors such as HPT, test temperature, and pain catastrophizing. HPT was also shown to be mainly influenced by individual environment experiences, and the test temperature was equal between twins. In another experimental study, heritability of pain catastrophizing has been estimated as 37% and individual environment as 63%, and has been shown to be directly related to experimental pain with a cold pressor task (61). Hence, the effectiveness of the conditioning procedure itself is affected by factors that are more attributable to individual environmental experiences than to genetic influences.

Twin studies are mainly performed to investigate and estimate the variance explained by heritability in diseases or symptoms, and the shared or common environment experienced by the twins within their family is considered to contribute to further similarity within twins, but the nonshared or individual environment component is considered a “residual term” (62), as it should contribute to dissimilarity. Turkheimer and Waldron (62) further elucidated the individual environment component and distinguished between objective and effective environment: even if the experienced objective environment can be the same, the effects on twins could be different. In our study all participants underwent the same conditioning procedure (objectively common), but the conditioning procedure was variably effective, and they responded in different ways to the placebo testing (effectively individual). This indicates interactional effects of genes by environment and complex interactions between common and individual environmental effects, e.g., how prior experiences shape subsequent experiences, which should be further investigated.

Finally, some limitations of our study should be mentioned and discussed. First, we did not assess zygosity through genetic testing, but relied on twins’ own information about genetic testing and questions about twin resemblance and dissimilarity. This procedure showed high consistency with genetic testing (27, 35, 36), but of course, it is not perfect. Second, we included male and female same-sex as well as opposite-sex twin pairs in our analyses, as female and male participants did not differ in pain-related outcomes. In contrast to our data, Roelke et al. reported significant sex differences in HPT but not in WDT (37), and sex differences in placebo analgesia through verbal suggestion were reported occasionally (5, 63). Therefore, sex differences should be further examined in subsequent studies with larger samples. Third, we report unadjusted *p* values for multiple testing for two reasons: 1) all predictors have been chosen reasonably based on previous results showing their association with placebo effects, and 2) we aim to stimulate further studies and assume that it is more helpful to report unadjusted p values. As *p* value adjustments are influenced by the number of tests performed as well as their significance levels, adjusted *p* values could be misleading for subsequent study design decisions about the inclusion of predictors. Fourth, the participants were blinded to the reduced temperature during the conditioning procedure, whereas our experimenters were not. Finally, in this experimental study, placebo analgesia was induced through conditioning with a well-established experimental paradigm in healthy volunteers to estimate the variance explained by heritability for the first time. Similar to experimental studies in general, results cannot be transferred to other situations without further research. Results should therefore be replicated in larger samples and with regard to other known placebo mechanisms such as verbal suggestions only and social learning, as well as with other experimental pain and other paradigms. Additionally, subsequent studies should estimate the variance in placebo effects explained by heritability in clinical samples, such as pain patients but also patients with other disorders.

In summary, we could show that heritability compared to the individual learning experience may play a minor role in placebo analgesia. However, interactions of genes and environment can still be a source of dissimilarity between twins; the search for candidate genes or polymorphisms is still important in the way to utilize placebo effects; and future studies should combine twin studies and genetic analyses. Furthermore, our results are restricted to placebo effects through conditioning on pain in healthy volunteers and should be replicated with regard to other mechanisms and symptoms as well as in patients.

## Data Availability

The raw data supporting the conclusions of this manuscript will be made available by the authors, without undue reservation, to any qualified researcher.

## Ethics Statement

All participants were included after written informed consent only and received monetary rewards for their participation in this study. This study was approved by the Ethical Review Board of the University of Tübingen (project No. 814/2015BO1) and was conducted in accordance with the Declaration of Helsinki.

## Author Contributions

KW and PE contributed the conception and design of the study. NM and A-KH performed the study and organized the database. EH, NM, A-KH, AS, and KW performed the statistical analysis and contributed to the interpretation of data. KW wrote the first draft of the manuscript. EH wrote sections of the manuscript. All authors contributed to manuscript revision and read and approved the submitted version.

## Funding

This work was supported by the German Research Foundation for KW (Deutsche Forschungsgemeinschaft, DFG, WE5658/2-1), and we acknowledge support by Deutsche Forschungsgemeinschaft and the Open Access Publishing Fund of the University of Tübingen.

## Conflict of Interest Statement

The authors declare that the research was conducted in the absence of any commercial or financial relationships that could be construed as a potential conflict of interest.
